# BARRIERS TO ACCESS HEALTH AND MENTAL HEALTH SERVICES AMONG SOUTH ASIAN OLDER ADULTS

**DOI:** 10.1093/geroni/igae098.0626

**Published:** 2024-12-31

**Authors:** Janki Shankar, Papiya Das

**Affiliations:** University of Calgary, Calgary, Alberta, Canada; Indo-Canadian Women’s Association, Edmonton, Alberta, Canada

## Abstract

Research on older immigrants in Canada is in its infancy despite their growing numbers. In 2019, Covenant Health, one of the largest healthcare providers in Canada, in partnership with the Indo-Canadian Women’s Association, a non-government service organization for new immigrants, in Edmonton, conducted a community consultation with South Asian older adults to understand their health and mental care needs and barriers to their access. The consultation was based on anecdotal evidence that South Asian older adults make poor use of health care services in general and mental health care services in particular. Focus groups and individual interviews with older adults were used to gather information, and the data were analyzed using thematic analysis. The results showed that a significant number of South Asian older adults lead marginalized and isolated lives within their community. Lack of language skills, transportation difficulties, poor knowledge of health care services, financial dependence on sponsors, and adherence to traditional beliefs about health and mental health services are some of the barriers to accessing services. Based on these findings, several recommendations are suggested to improve healthcare access for older adults.

